# Highly accurate sequence imputation enables precise QTL mapping in Brown Swiss cattle

**DOI:** 10.1186/s12864-017-4390-2

**Published:** 2017-12-29

**Authors:** Mirjam Frischknecht, Hubert Pausch, Beat Bapst, Heidi Signer-Hasler, Christine Flury, Dorian Garrick, Christian Stricker, Ruedi Fries, Birgit Gredler-Grandl

**Affiliations:** 1 Qualitas AG, Chamerstrasse 56a, 6300 Zug, Switzerland; 2 Bern University of Applied Sciences, School of Agricultural, Forest and Food Sciences HAFL, Länggasse 85, 3052 Zollikofen, Switzerland; 3 Chair of Animal Breeding, Technische Universität München, Liesel-Beckmann-Str. 1, 85354 Freising, Germany; 4 Agriculture Victoria, AgriBio, Centre for AgriBioscience, Bundoora, VIC 3083 Australia; 5 ETH Zurich, Tannenstrasse 1, 8092 Zurich, Switzerland; 6 Institute of Veterinary, Animal & Biomedical Sciences, Massey University, 4442 Palmerston North, New Zealand; 7 agn Genetics GmbH, 8b Börtjistrasse, 7260 Davos, Switzerland

**Keywords:** Whole genome sequencing, Imputation, Accuracy, Genome-wide association study, QTL discovery, Milk traits, Brown Swiss, Dairy cattle

## Abstract

**Background:**

Within the last few years a large amount of genomic information has become available in cattle. Densities of genomic information vary from a few thousand variants up to whole genome sequence information. In order to combine genomic information from different sources and infer genotypes for a common set of variants, genotype imputation is required.

**Results:**

In this study we evaluated the accuracy of imputation from high density chips to whole genome sequence data in Brown Swiss cattle. Using four popular imputation programs (Beagle, FImpute, Impute2, Minimac) and various compositions of reference panels, the accuracy of the imputed sequence variant genotypes was high and differences between the programs and scenarios were small. We imputed sequence variant genotypes for more than 1600 Brown Swiss bulls and performed genome-wide association studies for milk fat percentage at two stages of lactation. We found one and three quantitative trait loci for early and late lactation fat content, respectively. Known causal variants that were imputed from the sequenced reference panel were among the most significantly associated variants of the genome-wide association study.

**Conclusions:**

Our study demonstrates that whole-genome sequence information can be imputed at high accuracy in cattle populations. Using imputed sequence variant genotypes in genome-wide association studies may facilitate causal variant detection.

**Electronic supplementary material:**

The online version of this article (10.1186/s12864-017-4390-2) contains supplementary material, which is available to authorized users.

## Background

Different densities of genotypes can be derived from various SNP (single nucleotide polymorphism) chips [1, 2] or whole-genome sequencing approaches [3]. To combine genotype data from different densities, genotype imputation is required [4–7]. Genotype imputation infers missing genotypes in silico based on a reference population for which those genotypes are not missing [8]. In cattle, the 1000 Bull Genomes Project is a community-based approach to exchange next-generation sequencing (NGS) data of important ancestors of current cattle breeds [9, 10]. The run released in 2015 (Run 5) of the 1000 Bull Genomes Project includes 1577 *Bos taurus* and 115 *Bos indicus* genomes [10]. The analysis of sequence variant genotypes from the 1000 Bull Genomes Project facilitated to pinpoint causal mutations for monogenic traits [9]. For some animals, including a large proportion of Brown Swiss cattle (BSW), the accuracy of variant calling has been analyzed [11]. Imputation accuracy has been evaluated in Holstein, Jersey and Fleckvieh cattle using sequence variant genotypes from the 1000 Bull Genomes Project as reference data [12, 13].

The accuracy of imputation has been evaluated for various livestock species (e.g. cattle [4, 9, 12, 13], sheep [7], horses [6, 14] and pigs [15]). Most studies evaluated the accuracy of imputation from low- or medium density to a high density (HD) SNP chip panel (e.g [4, 5]). Accuracy of imputation from 50 k or HD to sequence level genotypes has also been evaluated in different species and breeds [6, 13, 16, 17]. Parameters affecting the accuracy of imputation include population and sampling structure such as the degree of relationship between validation and reference individuals [5, 6], and the quality of the reference genome [4]. However, the accuracy of imputation may vary along the genome; a regional decrease in imputation accuracy may result from misplaced SNPs [4]. Furthermore, the accuracy of imputation is low in regions where the genome contains large numbers of structural variants [13]. It has also been shown that step-wise imputation from 50 k to HD to sequence level genotypes yields higher accuracy compared to direct imputation from 50 k genotypes to sequence level [16].

An increased marker density may facilitate downstream analyses such as genome-wide association studies (GWAS) and genomic prediction may become more accurate [18, 19] because by imputing to whole-genome sequence variants, causal variants are likely to be included in the data set and they might be identified more easily [13, 20].

In this paper, we investigated the accuracy of imputation from HD to sequence-level genotypes in the BSW population. We used four imputation tools and different reference populations to determine the most accurate imputation approach for BSW. Subsequently, 1646 genotyped BSW bulls were imputed to sequence level to perform GWAS for lactation traits. Among the most associated SNPs we could identify variants that had been suggested to be causal in previous studies.

## Methods

### Whole-genome sequence data

**Fig. 1:**
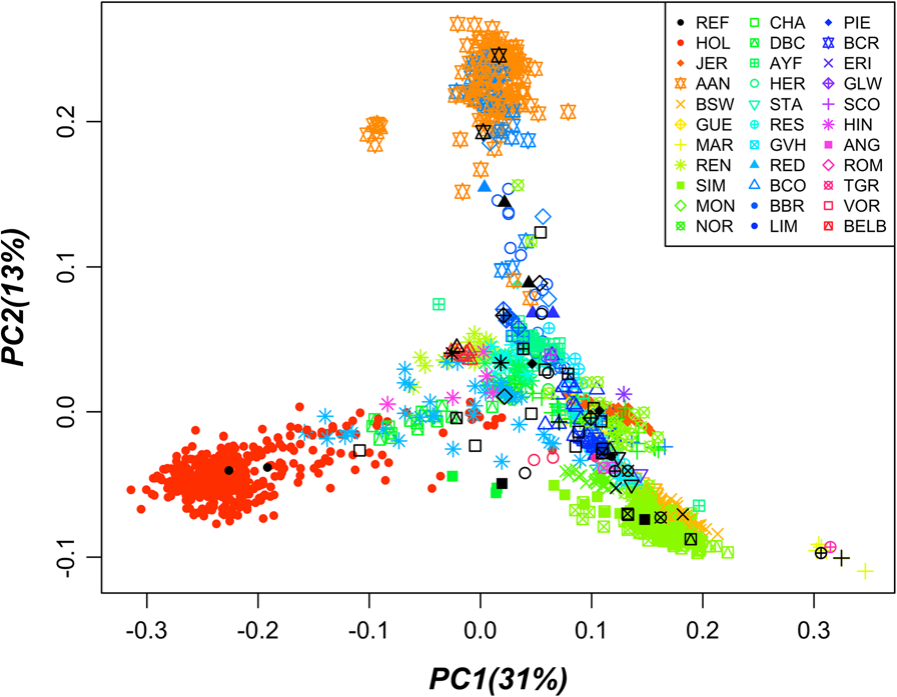
PCA plot showing the population structure of 1577 sequenced animals of the 1000 Bull Genomes Project Run 5. Different colours and symbols separate the animals by breed. Symbols in black colour represent indicate individuals selected as reference population in scenario S1 (REF in legend); HOL: Holstein; JER: Jersey; AAN: Angus; BSW: Brown Swiss; GUE: Guernsey; MAR: Marchigiana; REN: Norwegian Red; SIM: Simmental; MON: Montbeliarde; NOR: Normandes; UNK: unknown; CHA: Charolais; DBC: Dairy-Beef Crosses; AYF: Finnish Ayrshire; HER: Hereford; STA: Stabilizer; RES: Swedish Red; GVH: Gelbvieh; RED: Danish Red; BCO:Beef Composites; BBR: Beef Booster; LIM: Limousin; PIE: Piedmontese; SAL: Salers; BCR: Beef Crosses; ERI: Eringer; GLW: Galloway; ROM: Romagnola; SCO: Scottish Highland; TGR: Tyrolean Grey; HIN: Hinterwalder; ANG: Angler; VOR: Vorderwalder; BELB: Belgian Blue

Whole-genome sequence data were obtained from Run 5 of the 1000 Bull Genomes Project [9, 10]. That dataset used includes genotypes at 39,721,987 sequence variants for 1577 *Bos taurus* animals from 34 breeds (Additional file 1: Table S1) including 123 sequenced BSW animals. The population structure of this data set is shown in Fig. 1. The animals had an average coverage ranging from 2.2 to 44.5 sequencing reads. The reads were aligned to the UMD3.1 reference of the bovine genome using BWA –MEM (Burrows-Wheeler Aligner) [24] and variant calling was carried out using all available genomes simultaneously with samtools [21] as described previously [9]. Genotype calls were phased with BEAGLE version 4 (BEAGLE) [22, 23]. For our analysis, only bi-allelic SNPs were used for imputation.

For the principal component analysis (PCA) plot (Fig. 1) the genomic relationship matrix was calculated based on all autosomal sequence variants using PLINK (version 1.9) [24, 25]. The principal components of the genomic relationship matrix were subsequently calculated using the R princomp function [26].

### Imputation scenarios

We evaluated imputation accuracy for seven different scenarios. The aim of scenario one (S1) was to exploit the full range of genetic diversity represented by all breeds of the full Run 5 dataset. Therefore, in S1 we randomly selected one animal of each breed and country for the reference set (*n* = 49, Table 1). The remaining animals (*n* = 1528) were used as a validation set (Fig. 1). In scenarios two (S2) and three (S3) only the 123 BSW animals were used as reference and validation sets (Table 1). The reference animals for these two scenarios were selected according to the approach described by [27] to find key ancestors, which capture most of the gene pool of a given population. This analysis is based on mean pedigree relationship. The pedigree relationship was calculated based on a pedigree including 4491 BSW animals that included the 123 sequenced animals and their ancestors. Out of 123 sequenced BSW animals, the 20 and 50 most influential animals were identified and used as reference populations in S2 and S3, respectively. The validation set consisted of the remaining BSW animals of the total 123 sequenced BSW animals (103 and 73 BSW animals in S2 and S3, respectively). In scenario four (S4) random selection of 20 BSW animals was used to form the reference sets in a ten-fold cross validation (Table 1). In scenarios five (S5), six (S6) and seven (S7) the effect of single- versus multi-breed reference populations on the accuracy of imputation was studied in a ten-fold cross validation as well. In S5 the validation individuals of S4 were used as reference individuals (103 BSW animals). In scenario six (S6), in addition to the 103 BSW animals from S6, all animals from the main dairy breeds with breed code Holstein or Simmental were used as reference population (in total 855 animals). In S7 all Run 5 animals were used as reference set (1557 animals). The validation sets in S5, S6, and S7 consisted of 20 BSW animals randomly selected as reference in S4 (Table 1).

**Table 1:** Overview of imputation scenarios and number of reference and validation animals within scenario

Scenario	Reference	Validation
S1	49 animals out of Run 5	1528 animals out of Run 5
S2	20 BSW	103 BSW
S3	50 BSW	73 BSW
S4	20 random BSW	103 BSW
S5	103 BSW	20 random BSW from S4
S6	BSW + HOL + SIM (855 animals)	20 random BSW from S4
S7	All Run 5 animals (1557 animals)	20 random BSW from S4

### Imputation algorithms

We inferred missing genotypes using four imputation tools, namely BEAGLE (version 4.0) [22], FImpute (version 2.3) [28], IMPUTE2 (version 2.3.2) [29], and Minimac3 (version 1.0.12, referred to as Minimac) [30]. BEAGLE, IMPUTE2 and Minimac are population based methods, whereas FImpute exploits information from linkage disequilibrium and pedigree. However, FImpute was run in a population imputation setting only using called genotypes from the VCF files from the 1000 Bull Genomes Project as input data. For IMPUTE2, Minimac and BEAGLE the sequence variants were phased using BEAGLE. The reference and validation individuals were extracted from the 1000 Bull Genome project file using VCFtools [31] and BEAGLE and Minimac were run with default parameters. IMPUTE2 was run on the whole chromosome. BEAGLE and Minimac provide both allele dosage data with continuously distributed values for genotypes ranging from 0 to 2 and the most likely discrete genotype (i.e. 0, 1 and 2), whereas IMPUTE2 outputs genotype likelihood only and FImpute outputs discrete genotypes only. To transform allele dosage data from IMPUTE2 to discrete genotypes the program GTOOL (version 0.7.5) was used [32] applying the –G option. We did not want to have missing genotypes in our data. To achieve this the genotype likelihood threshold for calling a genotype was set to 0.3, because we have three genotypes that can potentially be called (namely 0/0, 0/1, 1/1). The sum of these three genotype probabilities should be one. In general, if none of the three genotype probabilities passes the threshold the genotype should be set to missing. Given the threshold of 0.3 must be surpassed by at least one of the three genotypes, no values were set as missing in this dataset.

### Imputation accuracy

Accuracy of imputation from the Illumina bovineHD SNP chip panel to whole genome sequence variants was investigated for chromosome 25. The genotypes of the validation animals were masked on BTA25 to mimic animals genotyped with the HD SNP chip. Imputation was then carried out from 12,222 SNPs (HD SNP chip) to 642,911 SNPs (whole genome sequence).

To determine imputation accuracy the sequence-derived genotypes were compared to the imputed genotypes in the validation animals. SNPs that were included on the HD SNP Chip were excluded for the assessment of imputation accuracy. Furthermore we did not consider SNPs that were monomorphic in both, the reference and the validation population. We assessed three accuracy measurements. The first was genotype concordance, which was calculated as the ratio of the number of genotypes that have the same alleles in the true and the imputed data set to the number of genotypes in total. For the correlation measurements, the imputed and the sequence derived genotypes were centered according to Calus et al. [33]. We assessed genotype correlation per individual and calculated the average per scenario. For the scenarios with cross-validation runs, we used the average of each scenario to calculate the average across all scenarios. For Minimac, BEAGLE and IMPUTE2 we additionally assessed allele dosage correlation. It has been suggested that the measure of choice for imputation accuracy should be dosage correlation as this is supposed to be independent of minor allele frequency (MAF) and because dosage is supposed to be less biased [33]. Since no allele dosage data can be obtained for FImpute, we calculate genotype correlation between true and imputed genotypes as well and use all three measurements for comparisons.

### Accuracy by MAF-classes

Accuracy of imputation was also evaluated according to the MAF of SNPs. The MAF for each SNP was calculated in the reference population for each scenario and were classified in 13 different frequency classes with a focus on low MAF classes using the following thresholds: 0, 0–0.025, 0.025–0.05, 0.05–0.075, 0.075–0.1, 0.1–0.15, 0.15–0.2, 0.2–0.25, 0.25–0.3, 0.3–0.35, 0.35–0.4, 0.4–0.45, 0.45–0.5. The boundaries of MAF classes were defined as greater than the lower boundary and less than or equal to the upper boundary (Additional file 1: Table S2). SNPs in the MAF class 0 were excluded from accuracy evaluation. Evaluation of imputation accuracy was then performed for each MAF class for the same measurements, as described above. For the evaluation all SNPs of a MAF class were put in a single vector and the correlations were obtained from comparing the vector of imputed SNP genotypes or dosages and the genotyped SNP genotypes or dosages. SNP genotypes were centered according to [33].

### Imputation of sequence variants for genome-wide association study

To test the ability to detect causal mutations with imputed sequence variant genotypes, two GWAS for milk fat percentage were carried out. The target population included 1646 BSW bulls of which 1432 and 214 genotyped with medium- (Illumina BovineSNP50, MD) and high-density genotyping arrays (Illumina BovineHD, HD), respectively. The chromosomal position of the SNPs corresponded to the UMD3.1 assembly of the bovine genome [34]. Mitochondrial, X-chromosomal, Y-chromosomal and SNPs with unknown chromosomal positions were not considered for further analyses. Standard quality control was carried out for the MD and HD dataset separately (MAF > 0.01 or missing genotypes < 0.1). After quality control, the medium-density genotypes of 1432 bulls were imputed to HD based on a reference population that consisted of 1056 BSW bulls with HD genotypes using FImpute [28]. The reference panel included 842 BSW bulls with HD genotypes that were not part of the GWAS target population. The final target population consisted of 1646 BSW bulls with (partly imputed) genotypes at 573,650 autosomal SNPs. Whole-genome sequence data were obtained for 128 BSW animals from Run 5 of the 1000 Bull Genomes Project [9] and two other sequencing projects [11, 35]. We considered 13,938,818 autosomal sequence variants with MAF > 1% that were an intersection of two variant calling pipelines [9, 36]. Haplotypes of the sequenced animals were inferred using BEAGLE [22] and served as reference to impute genotypes for 13,938,818 variants in 1646 target animals with (partly imputed) genotypes at 573,650 SNPs (see above) using Minimac [29].

### Phenotypes for association testing

Response variables for association testing were daughter-derived phenotypes for milk fat content at two stages (FC_early_: lactation days 8–12; FC_late_: lactation days 298–302) of the first lactation. Estimated breeding values for milk (MY) and fat yield (FY) were obtained for the 5-day intervals from routine breeding value estimation for milk production traits [37]. Phenotypes for fat content (FC) expressed as a percentage of milk yield for the 5-day intervals were calculated using $\mathrm{FC}=200\ \mathrm{x}\ \Big(\frac{{\mathrm{FY}}_{\mathrm{basis}}+0.5\ \mathrm{x}\ \mathrm{FY}}{{\mathrm{MY}}_{\mathrm{basis}}+0.5\ \mathrm{x}\ \mathrm{MY}}-\frac{{\mathrm{FY}}_{\mathrm{basis}}}{{\mathrm{MY}}_{\mathrm{basis}}}$), where FY_basis_ = 282.18463 and MY_basis_ = 7080.298. The mean accuracy of the EBVs was 0.95 (± 0.06). The correlation between breeding values for FC_early_ and FC_late_ was 0.26.

### Genome wide association studies

We considered 13,036,370 sequence variants with imputation r^2^ [29] >0.3 for association analyses. Association testing of each imputed sequence variant was carried out with FC_early_ and FC_late_ using the EMMAX software tool [38]. The mixed model fitted to the data by EMMAX included the overall mean, the allele dosage data of each variant in turn (continuously distributed from 0 to 2) as a fixed effect and a vector of additive genetic effects ~N(0,**G**σ_a_^2^) where **G** is the realized genomic relationship matrix that was constructed based on genotypes of 573,650 autosomal SNPs (see above) [39]. Sequence variants with *P* < 3.84 × 10^−9^ were considered as significantly associated (5% Bonferroni-corrected significance threshold for 13,036,370 independent tests). Bonferroni-correction might result in a too stringent significance threshold in association studies with imputed sequence variant genotypes because it assumes that individual tests are independent and is thus likely prone to over-correction considering the small effective population size and long-range LD particularly in livestock (e.g., Pausch et al., [40]). However, for this study we were mainly interested in major QTLs and the variants with the lowest *p*-values.

## Results and discussion

### Accuracy of imputation

We evaluated imputation accuracy for the BSW individuals included in Run 5 of the 1000 Bull Genomes Project [9, 10] in seven scenarios with different sets of reference and validation individuals (Table 1). Imputation from HD to sequence level was done on BTA25 using four different tools, namely BEAGLE [22], FImpute [28], IMPUTE2 [29], and Minimac [30]. We chose BTA25 for the validation because this is the smallest chromosome, which should consequently lead to the shortest computational times. It has been shown previously that there are differences in imputation accuracy per chromosome [6, 13]. We expect that similarly to [13], the imputation accuracy of BTA25 will not be significantly impaired by structural variation.

As measures of accuracy we evaluated genotype concordance rate and dosage correlation, as well as genotype correlation between called and imputed sequence variants.

### Scenario 1

In S1, the reference set consisted of 49 animals representing all *Bos taurus* breeds and breed groups from different countries included in the Run 5 data set (Fig. 1, black points). The average genotype concordance, genotype correlation and allele dosage correlation for BSW validation animals ranges from 0.953–0.972, 0.766–0.872, and 0.945–0.959, respectively (Table 2, Additional file 2: Fig. S1). The lowest genotype concordance rate is found using FImpute (0.953) whereas the highest rate of correctly imputed variants is found using Minimac (0.972). When evaluating all validation animals across all breeds jointly, slightly higher accuracies than for BSW animals only are observed. As for BSW animals only, the highest imputation accuracy is found using Minimac, however accuracies found using Impute2 are very similar. We find differences in imputation accuracy per breed (Additional file 1: Table S3). The lowest average imputation accuracy is achieved for Marchigiana and Tyrolean Grey cattle. The PCA plot indicates that the Marchigiana breed is different from most other breeds, which probably explains the low accuracy of imputation found in that breed. The accuracy in Tyrolean Grey is derived from a single individual that might be unrelated to the second Tyrolean grey in the data set, which is in the reference population, resulting in low accuracy of imputation.

**Table 2:** Mean (and standard deviation) genotype concordance, genotype correlation and allele dosage correlation for validation animals in S1

	Genotype concordance rate	Genotype correlation	Dosage correlation
BEAGLE BSW^a^	0.964 (0.004)	0.829 (0.019)	0.945 (0.011)
FImpute BSW	0.953 (0.005)	0.766 (0.021)	–
IMPUTE2 BSW	0.971 (0.004)	0.872 (0.021)	0.956 (0.009)
Minimac BSW	0.972 (0.004)	0.872 (0.021)	0.959 (0.008)
BEAGLE All^b^	0.967 (0.007)	0.840 (0.035)	0.965(0.010)
FImpute All	0.956 (0.008)	0.775 (0.038)	–
IMPUTE2 All	0.974 (0.007)	0.879 (0.035)	0.972 (0.009)
Minimac All	0.975 (0.007)	0.879 (0.035)	0.974 (0.008)

^a^Imputation accuracy evaluated for BSW validation animals only

^b^Imputation accuracy evaluated for all validation animals

### Scenarios 2–4

In Scenarios 2–4 we contrasted random selection of reference individuals (S4) to selection of key ancestors (S2 and S3). While S2 contains the same number of individuals as the random selection scenarios in S3 we selected 50 reference individuals. The idea of using key ancestors as reference animals according to [27] is that the selected individuals should be selected particularly to cover a large fraction of the genetic diversity of the entire population. The comparison of S2 and S3 also shows the impact of the number of individuals in the reference population. For S3 we find higher accuracies (Table 3) than for S2 with all measurements and all programs (e.g. 0.964 vs 0.978 with Minimac for allele dosage correlation). This finding is consistent with [4], where the reference individuals were selected with the same algorithm and increasing the reference population led to higher accuracy. A further interesting finding is that the variation between the accuracies of the validation individuals is decreased in S3 compared to S2 (Additional file 2: Fig. S1). For the imputation to HD data it has been found that differences in imputation accuracy between individuals became smaller when the size of the reference population increased [4]. Comparing S2 with 20 reference individuals to S3 with 50 reference individuals, we observe the same trend. Concerning the accuracy per program we find, that generally IMPUTE2 and Minimac perform similar and outperform the two other programs.

**Table 3:** Mean (and standard deviation or range for cross validation scenarios) genotype concordance rate (Gen Conc), genotype correlation (Gen Corr), and allele dosage correlation (Dos Corr) between called sequence variants and imputed variants for animals in the validation set in scenarios S2 to S7

Gen Conc	S2	S3	S4	S5	S6	S7
Beagle	0.947 (0.024)	0.967 (0.013)	0.945 (0.94–0.95)	0.966 (0.96–0.972)	0.979 (0.976–0.981)	0.984 (0.983–0.986)
FImpute	0.938 (0.024)	0.957 (0.012)	0.935 (0.93–0.944)	0.960 (0.953–0.965)	0.974 (0.971–0.977)	0.982 (0.98–0.984)
IMPUTE2	0.962 (0.02)	0.973 (0.008)	0.959 (0.956–0.963)	0.9712 (0.966–0.975)	0.981 (0.978–0.983)	0.985 (0.984–0.987)
Minimac	0.96 (0.02)	0.972 (0.008)	0.957 (0.955–0.962)	0.968 (0.963–0.973)	0.980 (0.978–0.983)	0.9852 (0.983–0.987)
Geno Corr						
Beagle	0.863 (0.053)	0.914 (0.031)	0.857 (0.848–0.87)	0.914 (0.899–0.923)	0.932 (0.923–0.941)	0.932 (0.924–0.94)
FImpute	0.827 (0.052)	0.879 (0.029)	0.819 (0.805–0.845)	0.894 (0.878–0.904)	0.916 (0.904–0.926)	0.923 (0.91–0.931)
IMPUTE2	0.905 (0.042)	0.930 (0.02)	0.897 (0.891–0.908)	0.9261 (0.915–0.932)	0.939 (0.93–0.946)	0.938 (0.93–0.945)
Minimac	0.899 (0.041)	0.926 (0.022)	0.8912 (0.886–0.902)	0.9162 (0.905–0.924)	0.9358 (0.927–0.944)	0.935 (0.927–0.943)
Dos Corr						
Beagle	0.951 (0.028)	0.973 (0.013)	0.956 (0.951–0.961)	0.977 (0.972–0.979)	0.980 (0.978–0.982)	0.982 (0.98–0.983)
FImpute	–	–	–	–	–	–
IMPUTE2	0.964 (0.023)	0.977 (0.009)	0.967 (0.963–0.971)	0.979 (0.975–0.98)	0.980 (0.978–0.981)	0.981 (0.979–0.982)
Minimac	0.964 (0.023)	0.978 (0.009)	0.967 (0.963–0.971)	0.9786 (0.975–0.98)	0.982 (0.98–0.983)	0.983 (0.981–0.984)

Comparing S2 to S4 we compared selection of key ancestors according to [27] to random selection scenarios. The imputation accuracies were above 0.93 for the genotype concordance and allele dosage correlation and above 0.80 for genotype correlations for both scenarios (Table 3). Contrasting S2 to S4 we find consistently higher accuracies for S2 with genotype correlation and concordance rate. This is expected and consistent with [4]. For the 1000 Bull Genomes Project most individuals have been selected according to [27] (see e.g. [9, 11]) to maximize the genetic variation captured within a population. Therefore, we might not see such large differences in the genetic contribution of an individual to the population of sequenced animals we investigated in this study. If we had sequences of random individuals from the corresponding breeds, we would expect a higher variability in the contribution to the genetic diversity and therefore we would achieve more different results between a random selection scenario and the selection strategy applied in S2 and S3.

Concerning the program best suited we find with these scenarios that using genotype concordance and allele dosage correlation again using Minimac and IMPUTE2 generally lead to almost equal highest accuracies.

### Scenarios S5 – S7

Scenarios S5-S7 are designed to investigate the effect of a multi-breed reference population on accuracy. Due to the setting of the validation set, the reference sets are thus considerably larger than for S1-S4 (Table 1).

For S5-S7 we find accuracies from 0.960–0.985 (genotype concordance), 0.894–0.939 (genotype correlation) and 0.977–0.983 (allele dosage correlation). This is indeed larger than the accuracies found in S2 and S4. Accuracy constantly increases when adding more individuals to the reference population. Furthermore, we find also for S5-S7 that the differences between the individuals are reduced (Additional file 2: Fig. S1, Table 3) as in S3. This could be an indication that not only the general accuracy is increased with more individuals in the reference population, but also the variance between the imputation accuracies of individuals decreases. On the other hand, there might be an upper limit for the possible accuracy given by the accuracy of the variant calling from sequence data, leading to the above-mentioned findings.

Interestingly, S3 leads to equal accuracies as S5. Also in S3 the reference panel is relatively large compared to the validation panel. This finding could hint that the selection of individuals according to [27] is beneficial for imputation accuracy in a sense that the upper limit of imputation accuracy is reached faster using this approach than random selection.

In a similar scenario it was found that using a multi-breed scenario imputation accuracy is only marginally increasing when a relatively large within breed reference is combined with data of another breed [41]. Multi-breed reference populations are likely to be beneficial to overall accuracy, when only a very limited number of within breed samples are available [6]. However also in large populations like Holstein, a higher accuracy has been found by using a multi-breed reference population [13].

### Accuracy by MAF classes

**Fig. 2:**
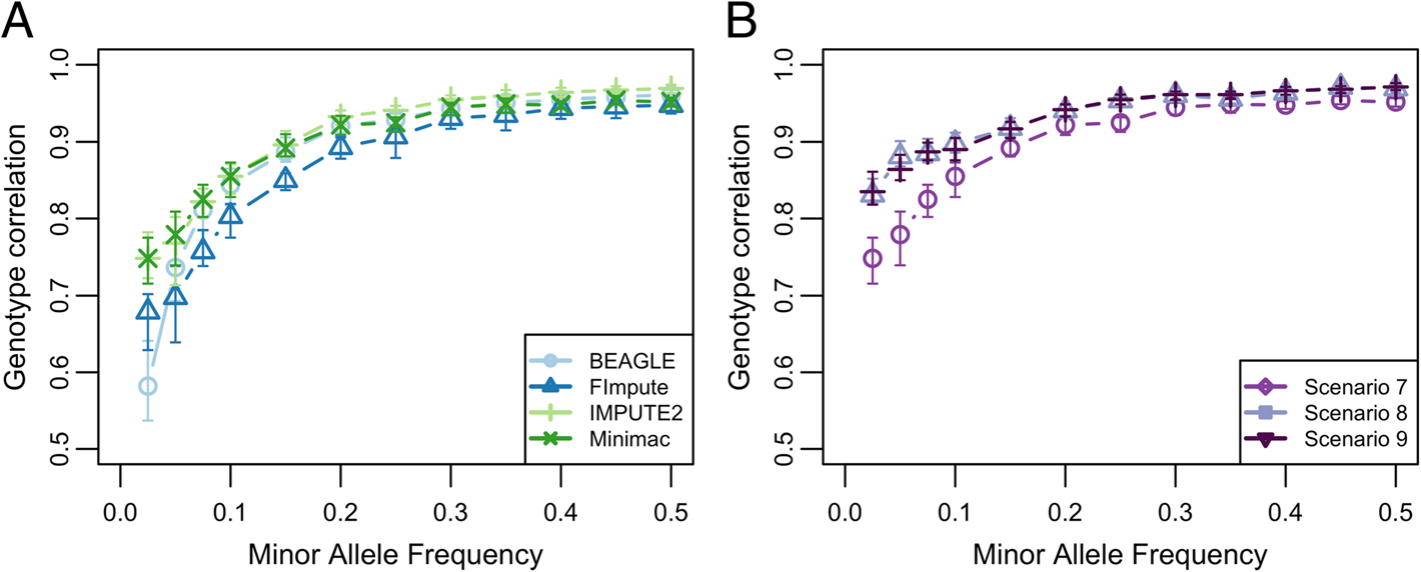
Genotype correlation by MAF class. **a** Genotype correlation by program. Mean genotype correlation (and range) obtained by imputation for each program (Beagle, FImpute, Impute2, Minimac) (**b**) Mean genotype correlation (and range) per MAF class with Minimac for S5-S7. The symbols are placed at the maximum MAF of the corresponding MAF class

For the scenarios S5-S7 we additionally evaluated the impact of MAF on accuracy of imputation, using dosage and genotype correlation. Generally we find that the higher the MAF the higher is the accuracy (Fig. 2, Additional file 1: Table S4). This is consistent with the findings of others (e.g. [41, 42]) and this is also the case for HD data [4]. Around a MAF of 0.25 the accuracy plateaus for dosage correlation and almost no further increase with higher MAFs can be observed. Looking at the different programs, BEAGLE shows the lowest accuracy for the lowest MAF class. The poor performance of BEAGLE for low MAF SNPs has also been found in other studies (e.g. [41]). However already in the second MAF class the accuracy from BEAGLE is higher than the accuracy from FImpute.

Comparing our different scenarios to each other we find that with dosage correlation all scenarios perform very similar across all the MAF classes, while with genotype correlation we observe the same trend as for the overall accuracy: The larger the reference population the more accurate is imputation. This finding is more pronounced from the BSW only to the dairy breeds and in FImpute, indicating that there might be an upper limit. Our findings indicate that a multi-breed reference panel may improve the accuracy of imputation for low-frequency variants. In another study it has been shown that low MAF variants only present within breed can be more accurately imputed with a multi-breed reference population [41]. Regarding the number of SNPs by MAF class (Additional file 1: Table S2) in the second smallest MAF class we find the largest number of SNPs for all scenarios. This is a typical finding when using sequence data and is different from SNP chip data [42]. For this reason it is also much more important to select a software tool that also imputes SNPs with low MAF with reasonable quality. In our study depending on the measure of accuracy and the scenario the tools Minimac and IMPUTE2 can yield very similar accuracy (Additional file 1: Table S4) and are therefore considered reasonable choices for imputation of sequence data.

### Association analyses with imputed sequence variants

**Fig. 3:**
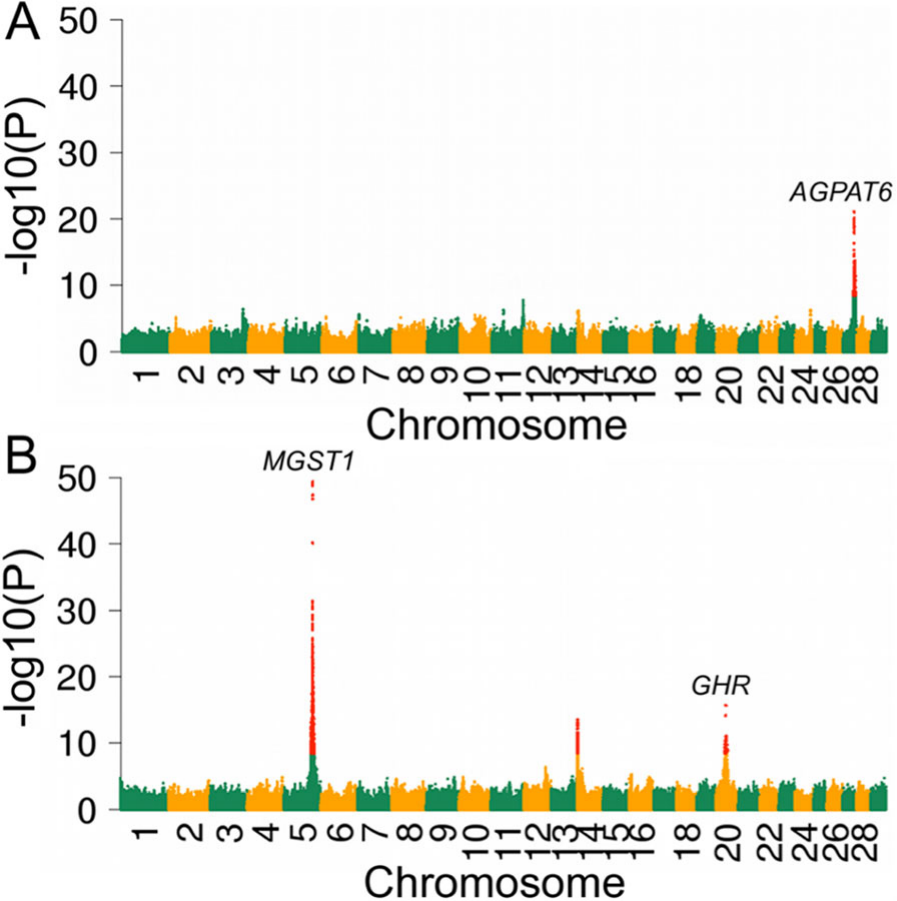
Identification of QTL for milk fat percentage at different lactation stages: Manhattan plot representing the association of 13,036,370 imputed sequence variants with fat content in early (**a**) and late (**b**) lactation. Red color represents variants with *p* < 3.84 × 10^−9^

More than 13 million sequence variants were imputed in 1646 BSW bulls that had (partly imputed) array-derived genotypes. To evaluate the precision of full-sequence data for quantitative trait loci (QTL) fine-mapping, we performed association studies with imputed sequence variants using daughter-derived phenotypes for milk fat percentage in early (FC_early_) and late (FC_late_) lactation as response variables. The inflation factors of the association studies were 1.05 and 1.10 for FC_early_ and late FC_late_, respectively, indicating that population stratification was appropriately considered. Association testing revealed one and three QTL (*P* < 3.84 × 10^−9^) for FC_early_ (Additional file 1: Table S5) and FC_late_ (Additional file 1: Table S6, Fig. 3). None of the QTL were associated with fat content at both lactation stages indicating a distinct genetic control of bovine milk production across the lactation cycle [43, 44]. The top association signals at all QTL result from imputed sequence variants demonstrating again the enhanced capacity of full sequence data for pinpointing candidate causal variants [45].

The top variant (*P* = 2.15 × 10^−16^) at a QTL for FC_late_ on BTA20 was a missense mutation (rs385640152, 31,909,478 bp, p.F279Y) in *GHR* encoding growth hormone receptor. The p.F279Y-substitution has been postulated to be a causal mutation for milk production traits in cattle [46]. Sequence-based association studies revealed that the p.F279Y- substitution is also highly significantly associated with milk production traits in Fleckvieh and Holstein cattle [47, 48]. Such findings are encouraging because they demonstrate that causal variants may be readily identified in association studies with imputed sequence variants. In our study, the GHR 279F variant enhanced FC_late_, which agrees with previous findings [20, 46]. In BSW, the frequency of the phenylalanine-encoding allele was 0.92, which is similar to the frequency observed in Holstein-Friesian and Fleckvieh cattle [20].

Another QTL for FC_late_ was located at the proximal region of BTA14. The top association signal resulted from an imputed sequence variant (ss1947221089, 2,179,252 bp, *p* = 3.33 × 10^−14^) in close neighborhood to a missense mutation in *DGAT1* (rs109326954, 1,802,265–1,802,266 bp, p.A232K) with a large effect on milk yield and milk composition in cattle [49, 50]. However, our association study indicated a variant other than rs109326954 to underpin FC_late_ in BSW cattle. The p.A232K-variant (rs109326954) in *DGAT1* was not polymorphic among the sequenced BSW animals of the present study corroborating its low frequency in the BSW population [50, 51]. However, the minor allele of the FC_late_ QTL on BTA14 had a frequency of 26.23%. Thus the association of ss1947221089 with FC_late_ does not result from linkage disequilibrium with the p.A232K-variant in *DGAT1*. Our findings indicate that a QTL other than *DGAT1* at the proximal region of BTA14 affects milk production in BSW cattle [44, 52, 53]. In our study, 399 imputed sequence variants located between 1,329,014 bp and 2,576,623 bp were significantly associated (*P* < 3.84 × 10^−9^) with FC_late_. Among them, a missense mutation (ss1947221094, 2,202,392 bp, *P* = 1.76 × 10^−13^, p.V307I) in *LOC506831*. However, many sequence variants had nearly identical *P*-values and pinpointing a putatively causal mutation was not attempted in our study.

**Fig. 4:**
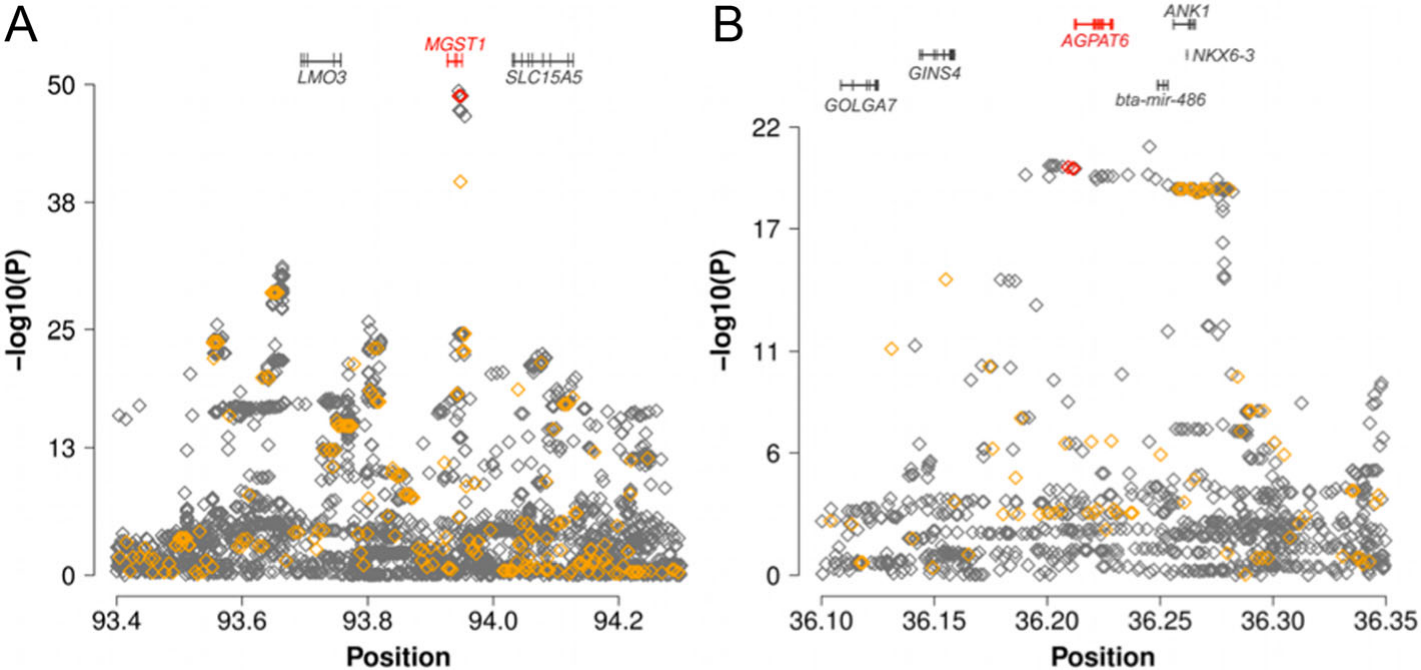
Detailed view of two QTL for fat content: Detailed overview of two QTL on BTA5 (**a**) and BTA20 (**b**) that were associated with FC_late_ and FC_early_, respectively. Grey and orange diamonds represent sequence and array-derived variants, respectively. Red diamonds represent candidate causal trait variants that were identified in breeds other than Brown Swiss

On BTA5, 20 variants located in the first intron and in the promoter region, respectively, of *MGST1* (microsomal gluthathione S-transferase 1) had P-values markedly lower than all other variants (Fig. 4a). The top variant (rs384016750, 93,944,908 bp, *P* = 4.34 × 10^−50^) resided 1369 bp upstream of the translation start of MGST1. Four variants (rs211210569, rs208248675, rs134637616, rs209372883) that were associated with milk and fat content in Holstein [48, 54] and Fleckvieh cattle [13, 47], were in high linkage disequilibrium (r^2^ > 0.97) with the top variant. Their P-values (*P* < 1.48 × 10^−49^) were only slightly higher compared to the top variant indicating that a common variant at that QTL is likely to control milk production traits in several cattle breeds.

A QTL on BTA27 was associated with FC_early_ comprising 122 variants with P < 1 × 10^−14^ resided within a 127 kb segment on BTA27 (36,155,097 bp - 36,282,137 bp). The top variant (rs384016750, 36,245,242 bp, *P* = 9.10 × 10^−22^) was located 16 kb downstream of the translation end of *AGPAT6* encoding 1-acylglycerol-3-phosphate O-acyltransferase 6 (Fig. 4b). The expression of *AGPAT6* is correlated with milk lipid content and reaches its maximum early in lactation [55]. Four candidate causal variants (rs211250281, rs378026790, rs211036538, rs209855549) for FC_early_ are located in the promoter region of *AGPAT6* [9, 56]. These variants were in high linkage disequilibrium (r^2^ > 0.89) with rs384016750. Their P-values (*P* < 1.09 × 10^−20^) were only slightly above the P-value of the top variant.

## Conclusion

Achieving high accuracy of imputation to whole-genome sequence level is possible in Brown Swiss cattle using the 1000 Bull Genomes reference population. According to two of the three measurements (dosage correlation and genotype concordance) Minimac is the best-suited program for imputation of this data, although all methods were generally adequate. The final imputation and subsequent GWAS of 1646 BSW revealed top SNPs in the sequence data, which are not on the SNP chip, including a well-known true causal mutation for milk production traits. Further some causal variants were among the top SNPs. This underlines that GWAS using imputed whole-genome sequence might facilitate the identification of new causal variants.

## Additional files

Additional file 1: Table S1. Breed codes and number of individuals per breed within the fifth run of the 1000 Bull Genomes Project. **Table S2.** Average number of SNPs per minor allele frequency class in S5, S6 and S7. **Table S3.** Imputation accuracy per breed in S1. **Table S4.** Imputation accuracy per minor allele frequency class in S5, S6, S7. **Table S5.** *p*-values and SNPs with Bonferroni corrected significant association for FC_early_. Chr is the chromosome and Pos is the position of the SNP, they correspond to UMD3.1. R2 is an imputation quality measure provided by Minimac and P is the p-value from the genome-wide association study. **Table S6.** p-values and SNPs with Bonferroni corrected significant association for FC_late_. Chr is the chromosome and Pos is the position of the SNP, they correspond to UMD3.1. R2 is an imputation quality measure provided by Minimac and P is the p-value from the genome-wide association study. (XLSX 169 kb)

Additional file 2: Fig. S1. Boxplots of individual animal accuracy of imputation for all scenarios measured as genotype concordance rate (A), genotype correlation (B), and allele dosage correlation (C) between called sequence variants and imputed variants. Straight lines represent the mean accuracy of imputation across all scenarios per program. (PNG 901 kb)

## Abbreviations

BSW: Brown Swiss Cattle
FC_early_: Fat percentage in early lactation (lactation day 8–12)
FC_late_: Fat percentage in late lactation (lactation day 298–302)
GWAS: Genome-wide association study
HD: High density
HOL: Holstein Cattle
MAF: Minor allele frequency
NGS: Next-generation sequencing
PCA: Principal component analysis
QTL: Quantitative trait locus/loci
S1-S7: Scenario 1 – Scenario 7
SIM: Simmental Cattle
SNP: single nucleotide polymorphism
