# Supplementary figures and images for: Highly accurate sequence imputation enables precise QTL mapping in Brown Swiss cattle
Source: BMC Genomics. 2017 Dec 29;18:999. doi: 10.1186/s12864-017-4390-2 (PMC5747239; doi:10.1186/s12864-017-4390-2)

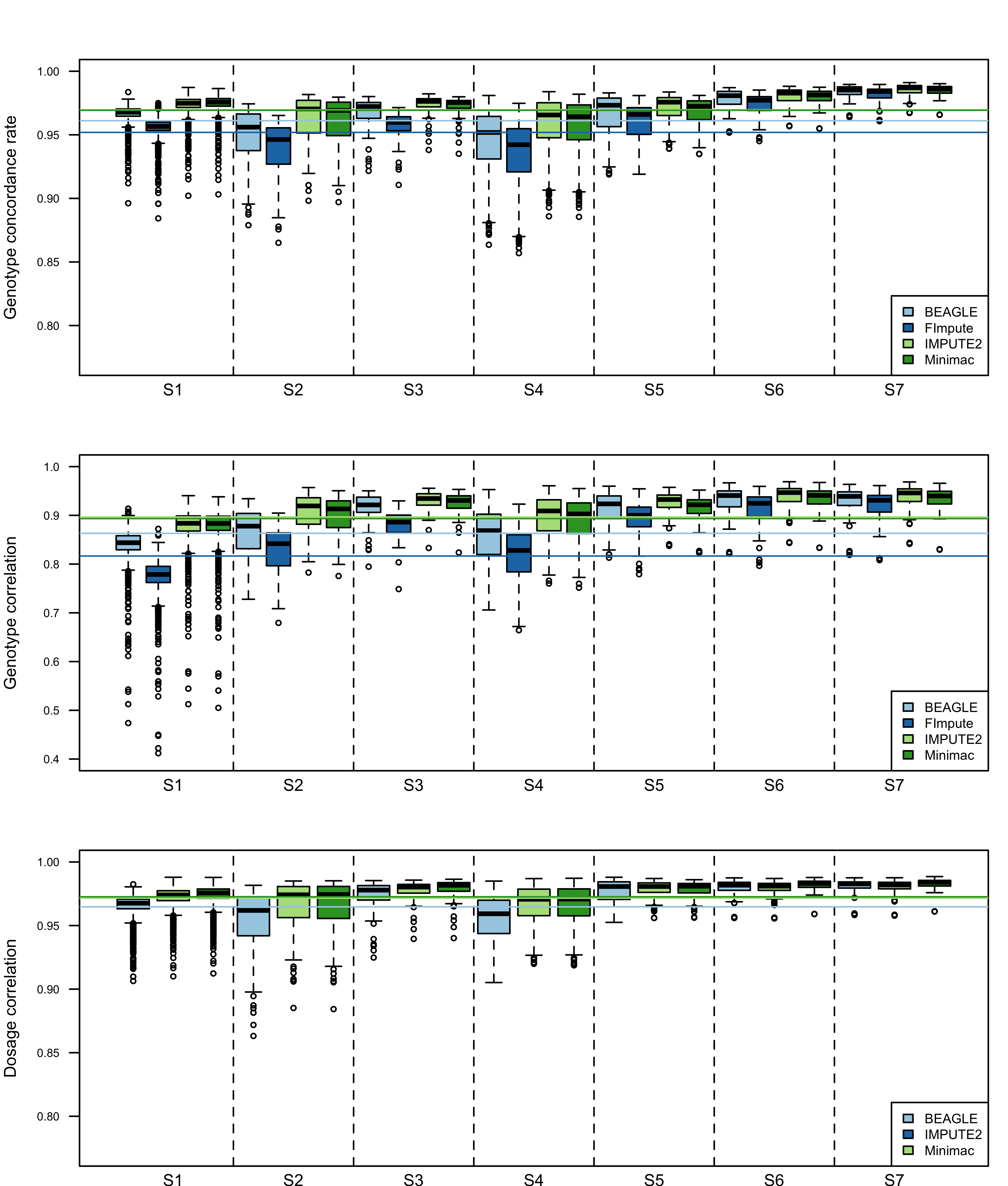

Supplement: Supplementary file 2 — Boxplots of individual animal accuracy of imputation for all scenarios measured as genotype concordance rate (A), genotype correlation (B), and allele dosage correlation (C) between called sequence variants and imputed variants. Straight lines represent the mean accuracy of imputation across all scenarios per program. (PNG 901 kb) [file 12864_2017_4390_MOESM2_ESM.png]
